# What does the French REIN registry tell us about Stage 4-5 CKD care in older adults?

**DOI:** 10.3389/fneph.2022.1026874

**Published:** 2023-01-17

**Authors:** Olivier Moranne, Aghilès Hamroun, Cécile Couchoud

**Affiliations:** ^1^ Service Néphrologie-Dialyse-Aphérèse, Hôpital Universitaire de Nîmes, Hôpital Carémeau, Nîmes, France; ^2^ UMR Inserm-UM, Institut Desbrest d'Epidemiologie et Santé publique (IDESP), Montpellier, France; ^3^ Service de Santé Publique, Service de Néphrologie-Dialyse-Transplantation rénale-Aphérèse, Hôpital Universitaire de Lille, Hôpital Huriez, Lille, France; ^4^ French REIN registry, Agence de la biomédecine, La Plaine Saint-Denis, France

**Keywords:** elderly, CKD, decision-making, registry, cohort

## Abstract

The aim of this paper is to illustrate all the clinical epidemiology searches made within the French network REIN to improve CKD stage 4-5 care in older adults. We summarize various studies describing clinical practice, care organization, prognosis and health economics evaluation in order to develop personalized care plans and decision-making tools. In France, for 20 years now, various databases have been mobilized including the national REIN registry which includes all patients receiving dialysis or transplantation. REIN data are indirectly linked to the French administrative healthcare database. They are also pooled with data from the PSPA cohort, a multicenter prospective cohort study of patients aged 75 or over with advanced CKD, monitored for 5 years, and the CKD-REIN clinical-based prospective cohort which included 3033 patients with CKD stage 3-4 from 2013 to 2016. During our various research work, we identified heterogeneous trajectories specific to this growing older population, raising ethical, organizational and economic issues. Renal registries will help clinicians, health providers and policy-makers if suitable decision- making tools are developed and validated.

## Introduction

The care of older people with chronic kidney disease (CKD) is a complex pathway that may benefit from personalized care plans and decision-making tools (1, 2).

Defining the older population is a complex issue in itself (3, 4). The medical literature reflects considerable heterogeneity in the definition of advanced age in research, although the threshold of 65 is most often used. However, medical advances and increased life expectancy are making those aged over 65 a very important part of the general population (5–7). The level of independence and the sheer proportion of this 65+ population are important considerations. Its level of autonomy and role in the active life of society raise the question of shifting the cursor to beyond the age of 75 (8). These debates remind us that the definition of the older population, currently limited to a criterion of chronological age, must evolve to include additional items relating to geriatric syndromes and frailty. For the purpose of this article, the word “older adults” will defined people aged 75 or over unless otherwise specified.

Indeed, older patients at an advanced stage of CKD are very heterogeneous in terms of clinical profile, functional, cognitive and medico-social status with various financial and emotional resources (9, 10). This subpopulation, more often than other CKD patients, presents alterations in these dimensions requiring specific global and personalized care (11). The therapeutic project will be made more complex with respect to renal replacement therapy, in particular a kidney transplant project (12–14). These frail patients will require multidisciplinary care for their kidney disease and their geriatric syndrome in a more medicalized structure. Their risk of death is high and should raise, even more, the question of the benefit of replacement therapy or the most appropriate therapeutic management (15, 16). In this context of the complexity of choosing the right therapeutic project with several options (i.e conservative care, dialysis, kidney transplantation), mixed quantitative and qualitative approaches are desirable to develop better specific support tools and improve the collegial decision-making process (17).

Moreover, the reduced life expectancy of these patients with Stage 4-5 CKD must be taken into account in organizing the care offer, especially access to supportive and end-of-life care (15, 18–20). Finally, the specificities of this population require a careful, though necessary, medico-economic approach (21).

Therefore, there is a definite need to describe and evaluate practices to improve global care and health management in this growing, changing population of older people with CKD (22–24). By providing data and indicators, registries are invaluable tools for following the evolution of care and may highlight variability in medical practices for advanced CKD like dialysis initiation, kidney transplant evaluation, modalities and location of treatment (25, 26). Registry data may also improve health system planning and policy-making by indicating efficient, cost-effective care strategies (27, 28).

The aim of this paper is to illustrate all the clinical epidemiology searches made within the French Renal Epidemiology and Information Network (REIN) over the last 20 years, in order to improve advanced CKD care in the older adults. We summarized various studies describing the clinical practice, care organization, prognosis and health economics evaluations in order to develop and improve personalized care plans and decision-making tools.

## Data sources

In France, the national REIN registry includes all patients receiving renal replacement therapy (RRT) - dialysis or transplantation - for End-Stage Kidney Disease (ESKD) (25, 29). Recording began in 2002 and the registry grew progressively to include the entire country (including overseas territories) in 2012. It aims to be a tool for public health decision support, evaluation and research. Anonymized data are available for researchers on request after validation by the REIN scientific committee. The data collection is ruled with an implicit consent with a drop-out option. Patients are informed on the purpose of the registry and the possible data linkage and data use *via* publicly available information (poster and welcome booklet in nephrology centers). Since 2019, the intention has been to expand it to patients with Stage 4 and 5 CKD not treated with RRT (30).

REIN data are indirectly linked to the French administrative healthcare database which consists of two main databases: the hospital discharge summaries database and the national health insurance claims database and it covers 98.8% of the French population, over 66 million people, from birth (or immigration) to death (31, 32). Analyzing this database provides insights into the economic burden of CKD and is a means to evaluate care consumption.

REIN was also linked to the PSPA cohort, a multicenter prospective cohort study of patients aged 75 years and older with advanced CKD monitored for 5 years. Details of the study design have been previously published and are summarized hereafter (33). Twenty-four French hospital nephrology centers were involved with an inclusion period of 4 months at each center. Inclusion criteria were age ≥ 75 years, CKD with eGFR < 20 ml/min/1.73m² (calculated by the Modification of Diet in Renal Disease Study equation) and seen by a nephrologist. Exclusion criteria were acute kidney injury and late referral for starting dialysis without any previous nephrology follow-up. The cohort included 573 patients with a median age of 85 years and a median eGFR of 14 ml/min/1.73m². The inclusion questionnaire asked about demographic data, clinical conditions (primary renal disease, comorbidities and disabilities), mobility (walking with or without help), place of abode and biological data. Nephrologists recorded the treatment plan at inclusion and at each subsequent visit during follow-up, depending on changes in their own opinion or the patients’ preference. The dialysis components of the treatment plans reported by the nephrologists were defined as follows (1): ongoing evaluation of the patient’s clinical condition and patient preferences after discussion (“Evaluation”) (2), postponement of the decision about dialysis due to stable clinical condition (“Stable”) (3); decision to start dialysis when it becomes necessary (“Dialysis”) (4), nephrologist’s decision that dialysis is inappropriate (“No Dialysis (Nephrologist)”) or patient’s decision against dialysis (“No Dialysis (Patient)”).

The study was approved by the Ethical Review Board of the research institution (Number 16.04.06), conducted in accordance with international clinical practice guidelines and registered on ClinicalTrials.gov under the number NCT02910908.

The CKD-REIN clinical-based prospective cohort enrolled 3033 patients with CKD stage 3-4 conducted in 40 nephrology outpatient facilities between 2013 and 2016 (34). Follow-up lasted 5 years, including after initiation of renal replacement therapy. The primary objective of the CKD-REIN cohort study was to develop a research platform to address key questions regarding various patient-level factors and biomarkers associated with CKD outcomes, and to assess clinical practices and healthcare system-level determinants of CKD outcomes. The study is registered on ClinicalTrials.gov (Identifier: NCT03381950).

**Figure 1:**
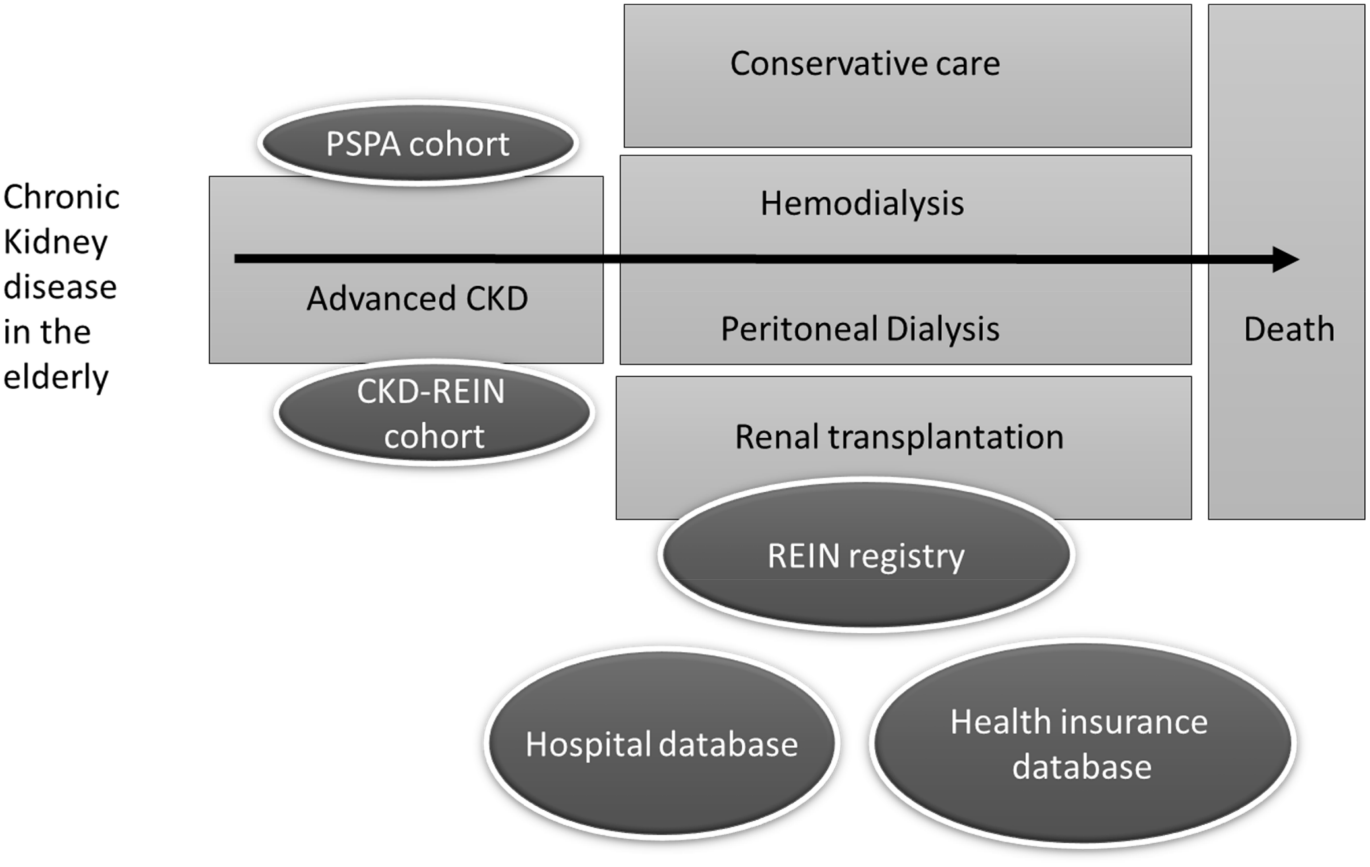
Data sources used.

With these datasets we were able to look at different trajectory points: advanced CKD care, transition to dialysis, transplant access, patient survival and overall care consumption (**Figure 1**). According to the outcome of interest, various statistical tools were used that are briefly described in the various paragraphs and detailed in each published study. Most of them took into account the competing risk of death.

## Results

### Advanced CKD: Complex, adaptive and multidisciplinary care

Baseline characteristics of the patients in the PSPA cohort suggest that the older patients referred to nephrologists are probably selected by their general practitioner and/or gerontologists (33). In fact, most of the patients are autonomous in walking and living at home.

These patients had a complex medical regimen with polypharmacy with an average of 9 medications prescribed per day (35). 77.0% of patients had at least one medication classified as a Renally Inappropriate Medication (RIM). At least one Potentially Inappropriate Medication (PIM) was taken by 57.6% of them and 45.5% of patients had at least one medication classified as RIM and PIM. In the PSPA cohort, the prescriptions most frequently requiring reassessment due to potential adverse effects were for proton pump inhibitors and allopurinol. The PIMs for which deprescription is especially important in this population are rilmenidine, long-term benzodiazepines, and anticholinergic drugs such as hydroxyzine. In the overall CKD cohort, anti-gout, cardiovascular agents and antidiabetic agents accounted for most of the inappropriate prescriptions (36). Standardized methodology of conciliation process of drug therapy applicable to these polypathological patients are needed (37, 38).

In this older population, healthcare providers must be more aware of the need to evaluate the benefit-risk ratio of each medication prescribed. Collaborative patient-centered approaches with all patients’ health care professionals must be developed. The development of new medication needs to consider this complex polypharmacy in this growing population.

The estimation of renal function itself appears to be a real challenge in older patients with CKD, mainly for two reasons: the first is that the recognized estimation formulas become inaccurate when renal function is impaired; the second is that most formulas have not been designed for the older population, especially those over 80 years of age (39–42). The Berlin Initiative Study (BIS) has shown that except Cockcroft–Gault equation, existing equations considerably overestimated GFR in older adults. They recommend the use of the BIS2 creatinine- and cystatin C–based equation to estimate GFR in persons aged 70 years or older with normal or mild to moderately reduced kidney function (39). The need for an accurate estimation of renal function is justified, in particular, by the need for dosage adjustment of prescribed drugs. In the study by Laville SM et al., the rate of inappropriate prescriptions in patients with CKD was particularly high, and depended directly on the estimation formula used (36). The CKD-REIN data emphasize the major prevalence of polymedication in patients with CKD: the patients included (median age 71 years, 2/3 over 65 years of age) received a median of 8 different therapeutic classes daily (36). In addition to polymedication, there is a significant risk of inappropriate prescriptions in this population, which is twofold: both with regard to age and the alteration of renal function, which justifies fine adjustments. Thus, half the patients were subject to at least one inappropriate prescription during their follow-up. The risk increased with the progression of CKD. Polymedication was also associated with a greater risk of serious adverse drug reactions, particularly frequent in this population (more than 14%/year), a third of which were considered preventable (43).

**Figure 2:**
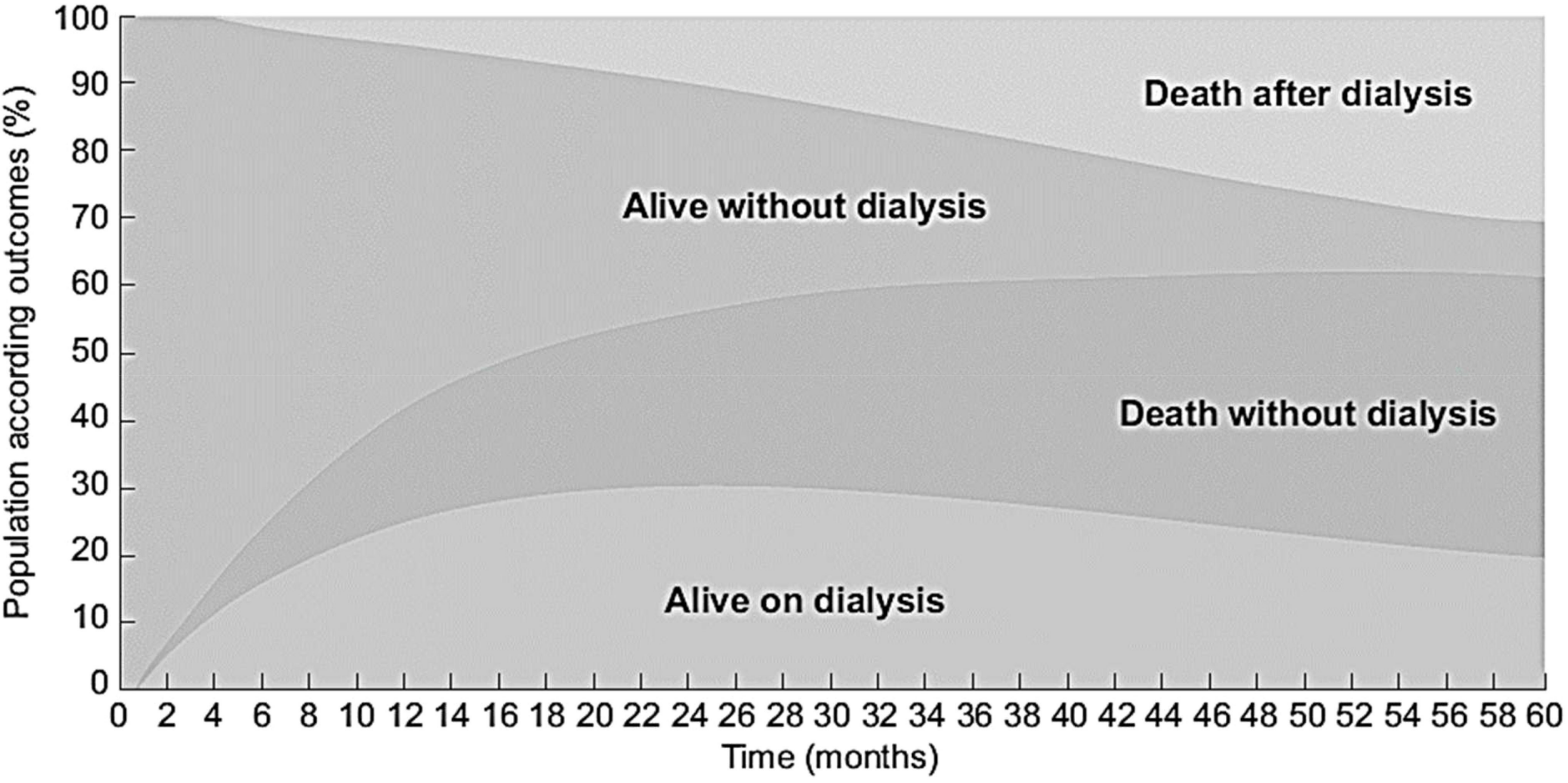
Evolution of the PSPA cohort with outcome according to time since inclusion for 573 CKD patients aged ≥ 75 years with eGFR < 20 ml/min/1.73m2 (source PSPA cohort linked to REIN registry). *Adapted from* (41, 44).

**Figure 3:**
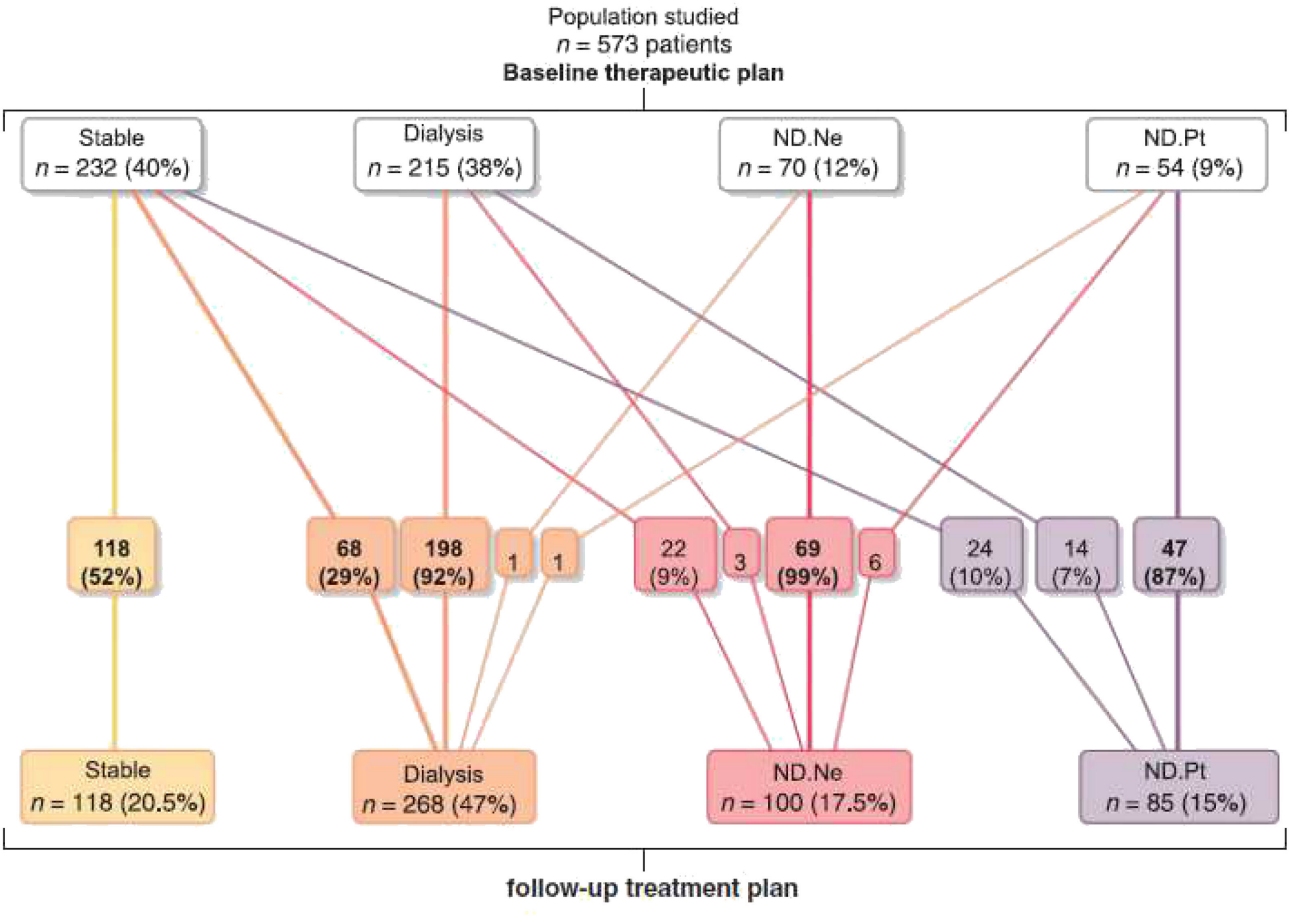
Evolution of treatment plans (source PSPA cohort). *Adapted from* (41, 44).

Although available and fully covered by the national health insurance for all who need dialysis, only half the older patients with advanced CKD (median eGFR of 14 ml/min) finally start dialysis and only a quarter were alive at 5 years (44) (**Figure 2**). This poor prognosis confirms the need to consider other perspectives such as quality of life and patients’ end-of-life wishes. However, older patients with very advanced CKD frequently do not articulate explicit decisions about dialysis until very late in their disease course. At baseline the treatment plans were “Stable” for 232 (40%) patients; “Dialysis” for 215 (38%); “No Dialysis (Nephrologist)” for 70 (12%), and 54 (9%) “No Dialysis (Patient)”. These treatment plans that may change over time were associated with their clinical condition and their outcomes (**Figure 3**). Determining a treatment plan early and re-evaluating it regularly will lead to better organization of patient care, whether it be dialysis or conservative management.

**Figure 4:**
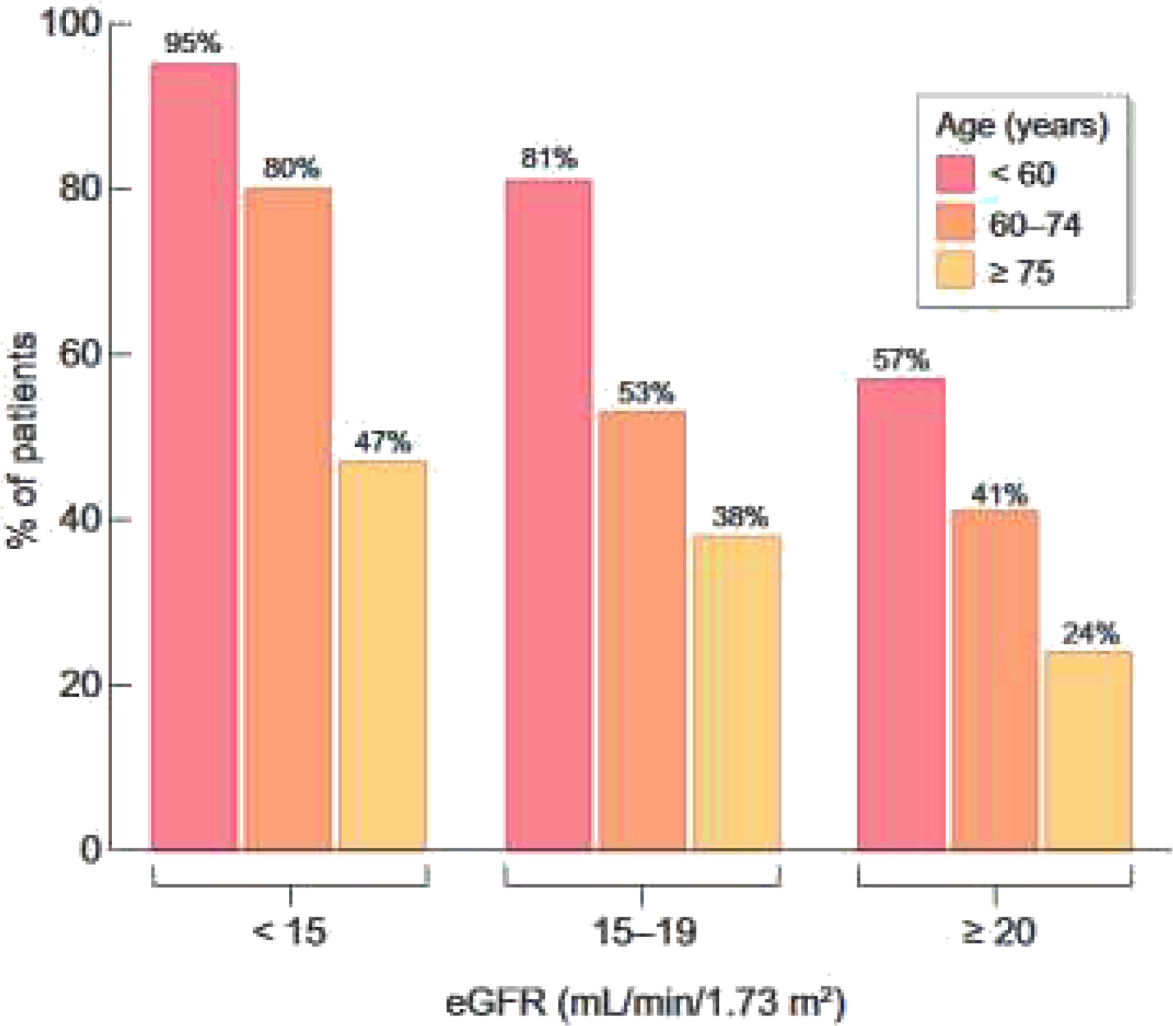
Percentage of patients who reported they have received education about kidney failure treatment options or have discussed them with their doctor, according to age and eGFR level. *Adapted from Hamroun et all 2022 (* 45).

In order to define a personalized care plan, a central place is given to clear, detailed information on treatment options. However, the data from France confirm that this information is provided very late in the course of CKD (if at all provided), and less often to older patients (**Figure 4**) (45).

The course of the treatment plan and the patient’s care trajectory upstream are essential information for evaluating dialysis initiation conditions as a quality criterion for medical practice. Because usual score have bad performance in this older population, specific prognosis scores before RRT are need that takes better into account competitive risk between progression to dialysis and death (46–48).

### Dialysis initiation: A high risk period

Although more medicalized due to the frequency of comorbidities, older patients are not protected from starting dialysis in an emergency (40% of the 2015 incident patients aged 75 years and over vs. 30% for the total population) (49). All effort should be made to prevent such situation. Patients with a lesser or lack of follow-up with a nephrologist are more likely to start dialysis in emergency, regardless of the frequency of follow-up by a general practitioner (49). Various strategies can be implemented to avoid late referral for specialized nephrology care. Even if early referral does not totally prevent urgent start, a planned approach in which the modality has been chosen prior to the need for dialysis and there is an access ready for use at the initiation of dialysis, is preferable. Many studies have shown poor outcomes of such urgent starters but also the use of temporary vascular access (50, 51).

**Figure 5:**
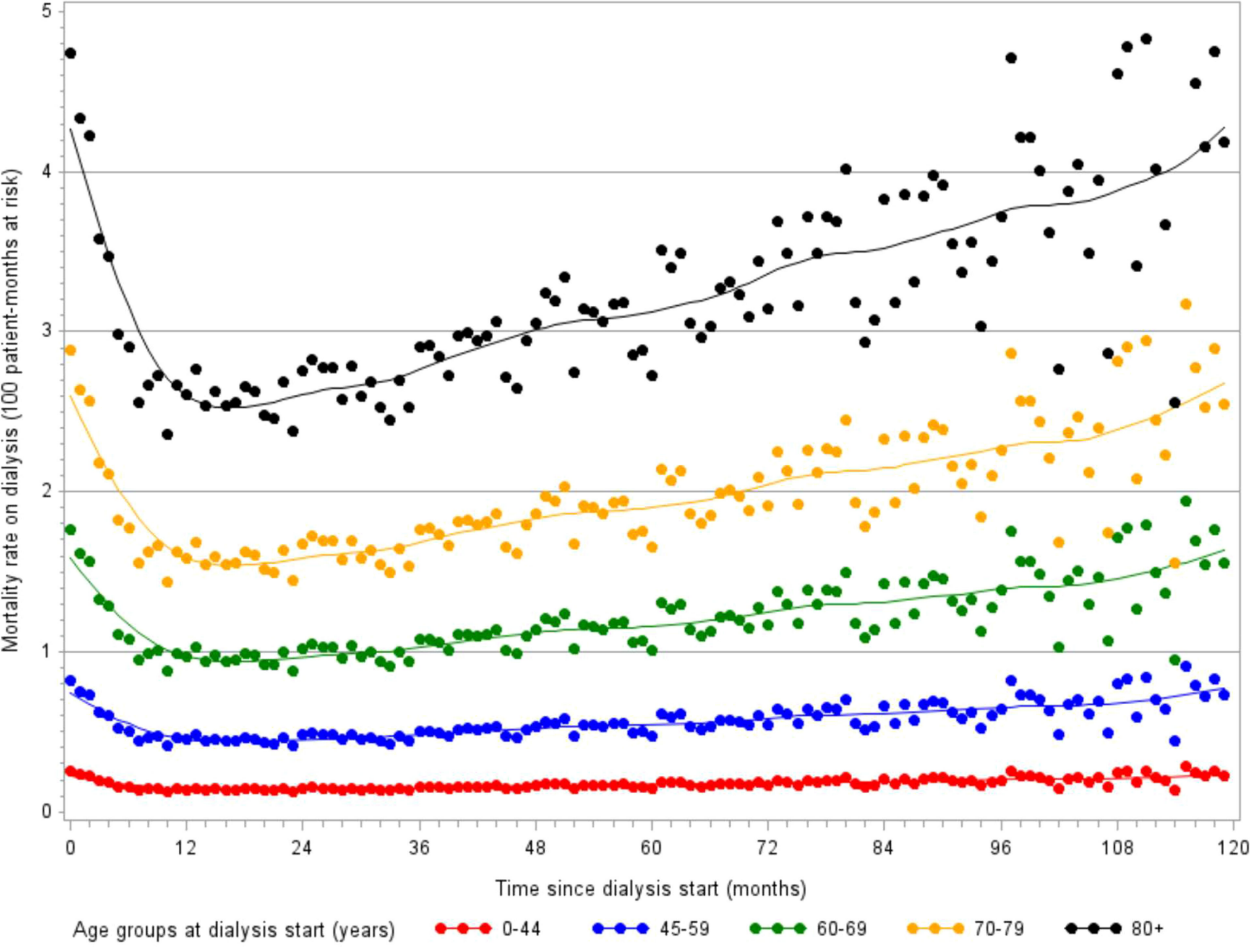
Evolution of monthly mortality rates over 10 years following dialysis initiation, stratified by age (monthly incidence estimated with a Poisson model, source REIN registry).

Among patients aged 75-84 years and 85 years and over, respectively 10.3% and 15.2% die within the first three months (52). This initial high mortality risk is followed by a strong decrease during the first year, but the mean mortality rate rises steadily over time on treatment (**Figure 5**). At dialysis initiation, 5% of the old patients are unable to walk without help, indirect item chose in the REIN registry to capture frailty. As well, 10% have sign of malnutrition defined as a serum-albumin level of <3 g/dl and/or a BMI of <20 kg/m2. This early mortality should lead to discussion about the complications related to dialysis management as well as the expected benefits in this fragile older population with no improvement on dialysis (15, 53). In this context, developing a prognostic score for early mortality after dialysis initiation can be interesting to inform patients and their relatives and help physicians in the difficult shared decision-making process to define the most appropriate therapeutic choice.

**Figure 6:**
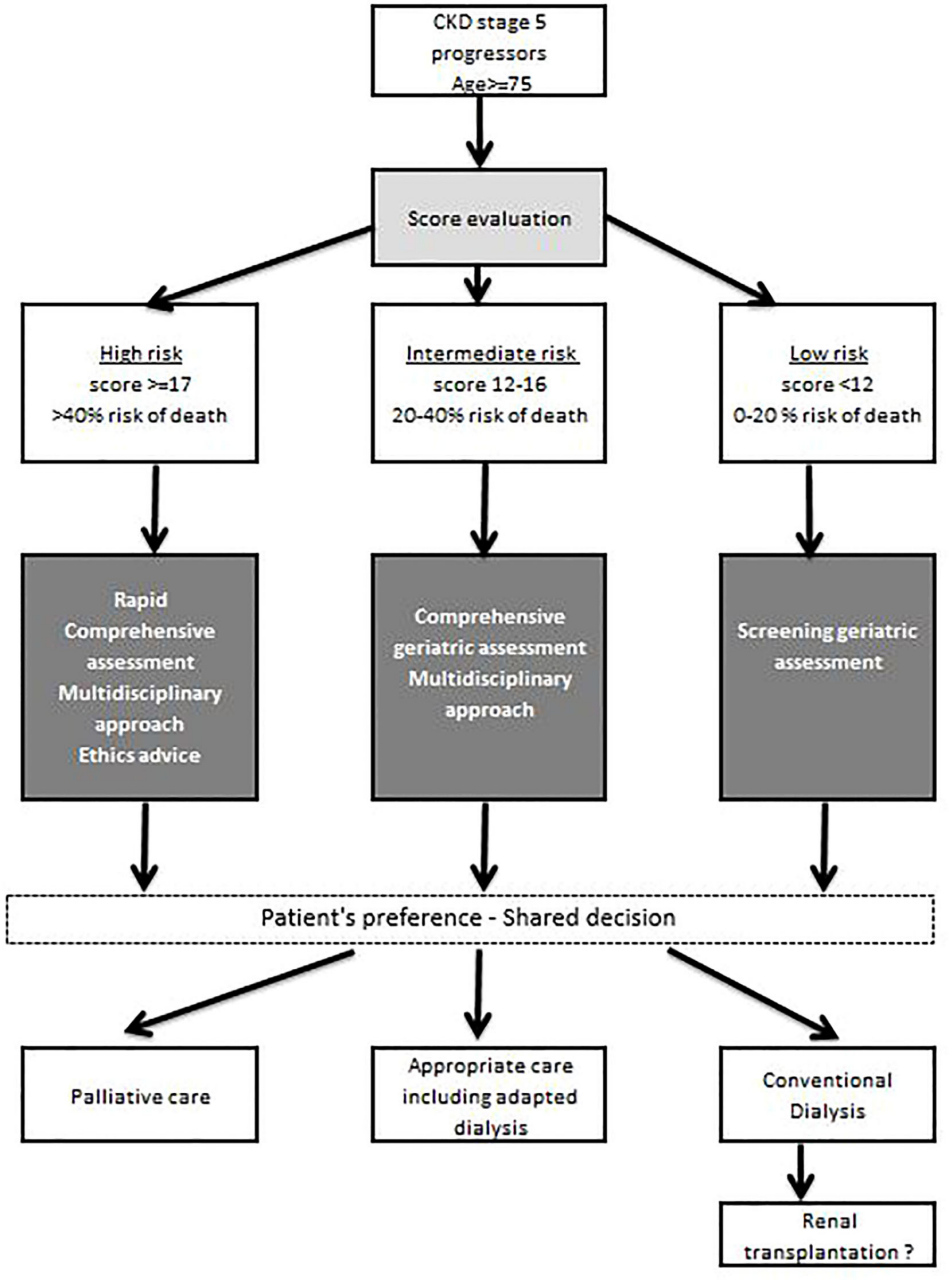
Proposed risk stratification algorithm to screen for, evaluate and decide on an appropriate care strategy for older ESKD patients, according to their level of risk of early death (source REIN registry). *Adapted from* Couchoud et al., 2015 (54).

A prognostic score for early mortality was therefore developed with REIN to make it possible to identify those patients at a low, intermediate or high risk of dying soon after starting dialysis, at the bedside or during a medical visit (54). High-risk patients may benefit from a multidisciplinary approach and more specific geriatric tools to better appreciate their functional status, comorbidities, cognition, mental health status, fatigue, social status and support, nutrition, and presence of other geriatric syndromes (**Figure 6**). The deployment of such a process should be accompanied by strengthening the cooperation between nephrologists, geriatricians, general practitioners, social workers and physicians trained in palliative care and by improving staff training in ethics and communication. The CKD-REIN cohort has shown that conservative treatment is only occasionally offered to older patients and most of them report that they were not informed of this option (45). In fact, only 28% of nephrologists report proposing this alternative to their patients aged 75+ at that stage of renal failure, and only 5% of patients report having heard of a treatment option without dialysis (**Figure 4**).

The REIN plans to extend data collection to Stage 4 and 5 CKD patients (30). A better understanding of CKD care and patients’ trajectories will enlighten nephrologists about the characteristics of the population selected to start renal replacement therapy. Difference in propensity to accept for dialysis older patients or with poor condition or with higher EGFR may explain geographical variation in ESKD incidence (55, 56). In older patients the median eGFR at dialysis start is at 10.0 vs 8.4 ml/min/1.63m² in patients age lower than 75 years.

### Low quality of life

**Figure 7:**
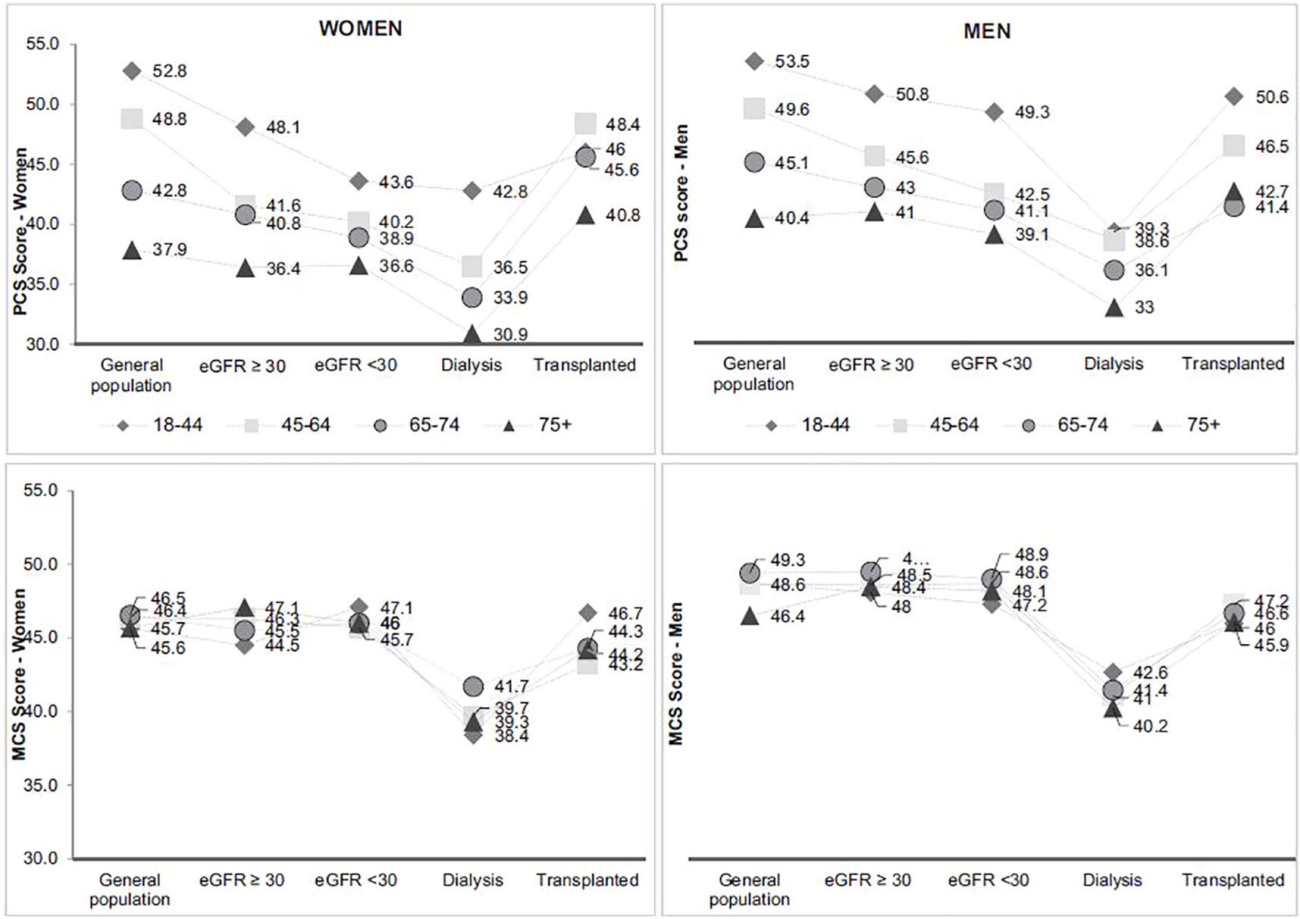
Physical and mental health score according to age and CKD status* (source CKD-REIN, REIN registry). *Adapted from Legrand et al., 2020* (57). MCS Mental Component Summary, PCS Physical component Summary. *Gray lines connect the symbols for each age group to facilitate visualization of each group between kidney disease status groups. They do not reflect the course of health-related quality of life across CKD.

As in the general population, the prevalence of fair or poor health status increases with advanced age in dialysis patients (57). However, in CKD and transplanted patients, patients aged 75 years and over do better than those aged 65-74 years old. In men, the prevalence of fair or poor health status in transplanted patients is similar in the 75-and-over age group compared to the general population with same age. Physical and mental health scores are lower in older patients in all populations except for the mental score in transplanted men (**Figure 7**). In all age group, PCS is decreasing with advanced CKD and dialysis and in a lesser extent in transplanted patients. Like in other studies, these results illustrate the clinical impact of CKD stages on the physical dimensions of Qol, especially in olders (58, 59). Differences are low in MCS score between the general population and the different stage of CKD except in dialysis illustrating the fact that only dialysis affects its mental dimensions. However, some studies have shown that the impact of CKD on Qol is likely multifactorial and partly mediated by co-occurent conditions (60).

Finally, the overriding objective of preserving quality involves adapting treatments to the needs and demands of patients that may evolve over time.

### Various suitable dialysis modalities in view of heterogeneous needs

In this older population, variabilities in treatment access can be observed. In a study aimed at identifying factors associated with choosing peritoneal dialysis as a first treatment, we found great variability between districts according to age (61). Higher age is associated with less home-based dialysis and increased mortality rates (62). It is associated with fewer switches to facility-based HD in PD patients but higher rates of switching from home-based HD to facility-based HD. In France, easier access to nursing-assisted care or help from a caregiver/family member for PD rather than home-based HD in older patients may explain this longer survival technique. Despite the lack of data on such practices, one can hypothesize that nursing-assisted PD may allow nephrology teams to keep frail older patients at home “until the end”. Moreover, the quality of life associated with home therapy may play a major role in the older patient’s choice of supportive care. Globally, home dialysis use as first treatment is low in France. While it remains stable around 5.6% for the patients aged lower than 75 years, it is decreasing in older patients (6.1% in 2012, 4.9% in 2020).

Indicators are especially difficult to interpret when the underlying dynamic process is not well understood. Therefore, we have developed a statistical tool to study the 10-year course of incident ESKD patient cohorts and to quantify, by simulations, the impact of various expected changes or new strategies (63). This tool is accessible to all French nephrologists on a secure portal.

### A complex situation for discussing access to kidney transplants

Because transplantation may be associated with better Qol, this treatment option should be systematically considered. However to improve kidney transplantation equity, one must empowering clinicians with adequate predictive tools.

The broadening of the indications for transplantation allowed by the use of extended criteria donors has prompted professionals to offer transplants to increasingly older patients. However, despite national and international guidelines, many studies have shown variations in kidney transplant waiting list practices or policies. Some guidelines define age limits whereas others consider that patients should not be deemed ineligible based on their age (64). Indeed, with older patients, the great heterogeneity of prognoses may complicate the careful assessment of eligible patients.

**Figure 8:**
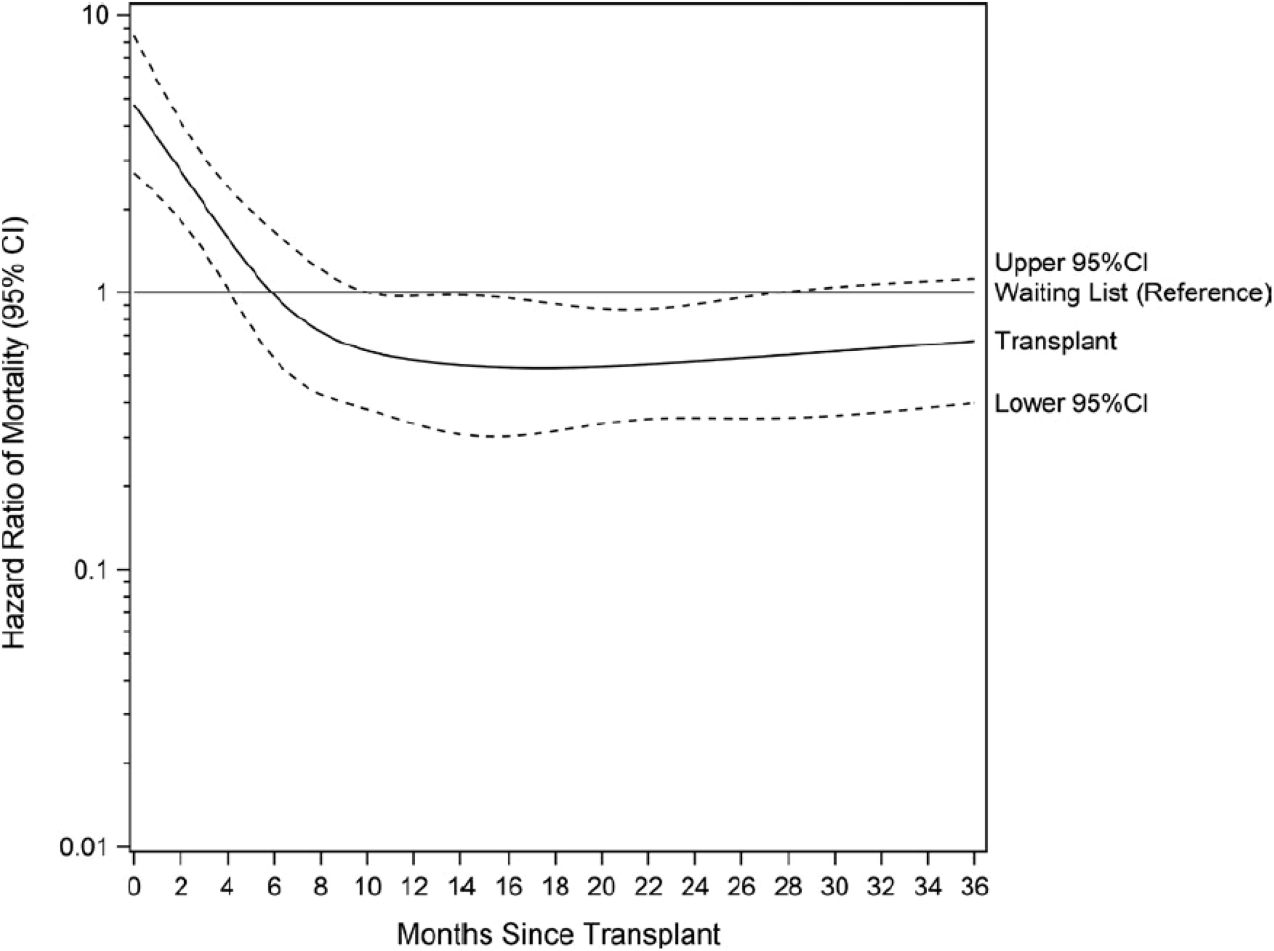
Relative risk of death after renal transplantation in older patients compared to wait-listed patients (source REIN registry). *Adapted from Legeai et al., 2018 (* 69 *)*.

Although available and fully covered by the national health insurance for all who need renal transplantation, in France, less than 2% of older patients (75 years and over) have access to the waiting list for a kidney transplant at 24 months (52). In a study aimed at identifying sources of variation in early kidney transplant waiting list registration in France, older age was associated with a lower probability of registration and greater variability between networks (65). Such lower access to registration for older patients has been shown in many countries (66–68). While the recommendation is to avoid setting a cut-off age limit for eligible older kidney transplant candidates without medical contraindications, it has been observed that there is a higher risk of death in the perioperative and early post-transplant period in this older population compared to wait-listed dialysis patients of similar age and cardiovascular risk (69) (**Figure 8**). These results lead us to recommend the systematic screening of all older patients for transplantation eligibility. The first screening is followed by a more in-depth assessment that takes into account their comorbidities, frailty index and the balance between the expected benefits and the risk of early death, which the patient must be informed of. This screening should be conducted as early as possible to rapidly identifying suitable candidates, limit the duration of dialysis or enable preemptive transplantation (70).

Given the median age of 71 years and the heterogeneity of prognoses of the incident cohort arriving at Stage 5 CKD each year, a decision tool capable of identifying those with a good long- term prognosis among all incident CKD patients could be an opportunity to help screening.Indeed, a kidney transplant project should be discussed with the patients and their relatives. An impact study is ongoing in France to evaluate the benefits of using a decision-making score to identify a subgroup of patients with a good long-term prognosis, as early as possible after starting dialysis. This could help nephrologists and older adults at the dialysis center to consider kidney transplant evaluation in collaboration with transplant centers (71).

### High hospitalization rates and care costs

In 2019, based on hospital database-REIN linkage, we were able to show that hospitalization rates increase with age (**Table 1**). They represent 1.5 hospitalizations per 1 person-year in patients age 75 years and over and 20% of these are related to cardiovascular disease and cardiovascular events (**Table 2**) (72).

**Table 1:** Hospitalization rates according to age (source REIN registry linked to hospital database).

Age group (years)	Number of patients at risk (PAR)	Number of full inpatient care	Full in-patient care/100 PAR	Number of days/100 PAR	Number of days in hospital	Number of days in hospital/100 PAR
00-19	256	496	194	1 414	733	287
20-44	3 994	4 020	101	626	4 036	101
45-64	15 108	16 829	111	783	18 127	120
65-74	14 803	21 030	142	1 023	21 436	145
75-84	13 900	21 371	154	1 106	16 384	118
85+	6 222	9 701	156	1 145	6 209	100

Adapted from Mercier et al., 2022 (72).

**Table 2:** Cause of hospitalization according to age (source REIN registry linked to hospital database).

CAUSES	Age (years)											
	00-19		20-44		45-64		65-74		75-84		85+	
	n	%	n	%	n	%	n	%	n	%	n	%
CARDIOVASCULAR	50	4.1	707	8.8	4 982	14.2	7 094	16.7	7 558	20.0	3 044	19.1
UROLOGY NEPHROLOGY	598	48.6	1 753	21.8	5 369	15.3	5 956	14.0	5 058	13.4	2 219	13.9
VASCULAR ACCESS	70	5.7	806	10.0	3 557	10.2	4 205	9.9	3 995	10.6	1 509	9.5
CANCER	5	0.4	125	1.6	3 735	10.7	5 605	13.2	2 977	7.9	751	4.7
INFECTION	68	5.5	345	4.3	1 605	4.6	2 051	4.8	2 284	6.0	1 208	7.6
ANEMIA	17	1.4	253	3.1	1 435	4.1	2 427	5.7	2 273	6.0	1 342	8.4
TRANSPLANTATION	58	4.7	503	6.1	1 046	3.0	542	1.3	162	0.4	1	0.0
OTHERS	363	29.5	3 564	44.2	13 227	37.8	14 586	34.3	13 447	35.6	5 836	36.7

Adapted from Mercier et al., 2022.

A health economic evaluation was performed based on the REIN and the National Health Insurance Database, to compare the efficiency of different care strategies. The overall mean monthly cost spent for a stable patient increases with age (27, 73). These higher costs are mainly explained by the higher costs of the dialysis treatment modality: hospital-based HD or nurse- assisted home APD (73). Alternative strategies, like the development of kidney transplantation from deceased donors with perfusion machines, the use of assisted CAPD or out-center HD could be more efficient than the current practices for certain patients (27).

### Low access to palliative care despite high mortality rates

**Figure 9:**
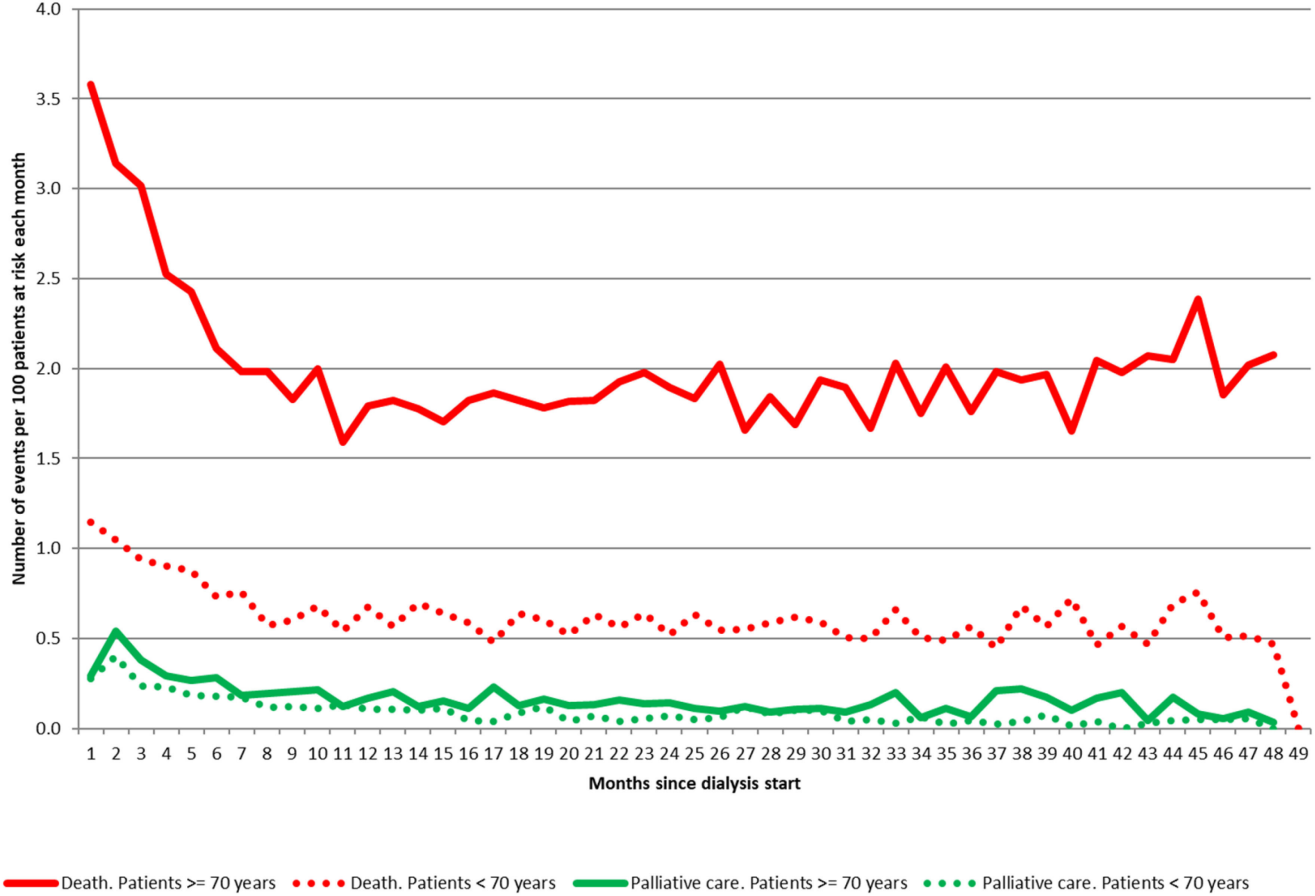
Monthly incidence rates over time of hospitalizations associated with palliative care and death from all causes during the first four years after starting dialysis, according to age (source REIN registry linked to hospital database). *Adapted from Couchoud et al., 2017* (74).

In a French cohort of 51,834 incident ESKD patients, 33% died during the study period, but only 3.6% of them resorted to palliative care-related hospitalization (74). Hospitalizations were classified as being “for palliative care” when the principal diagnosis at discharge was coded as Z51.5 (“Palliative Care” in the International Classification of Diseases, Tenth Revision, Clinical Modification). Hospitalizations classified as being “with palliative care” were defined by at least one night in a bed dedicated to palliative care during hospitalization, independently of the principal diagnosis. Older patients did not have more access to palliative care despite their higher mortality rate (**Figure 9**).

In this same cohort, only 10% of the patients who withdrew from dialysis (a situation in which death is expected and predictable) had palliative care-related hospitalization, compared to 43% of hospices used in the US in the same context. Only 19% died at home whereas many people with a terminal illness would prefer to die at home (75). Active cancer was the principal factor associated with the likelihood of palliative care hospitalization. This may reflect the current practice of acceptance in palliative care units or the nephrologists’ perceptions that palliative care is reserved for patients with cancer.

In the REIN registry, when declaring death, it is possible to indicate whether the treatment was discontinued and, if so, the reason why. From 2002 to 2019, 14,600 deaths (19%) occurred after withdrawing dialysis, with a median of 6 days (interquartile range: 3-13) (52).

## Prospects for renal registries and research

During these various research works, we identified heterogeneous trajectories specific to this growing aging population, raising ethical, organizational and economic issues **Table 3**. Therefore, decision-making tools must be developed and validated for this new specific population to help clinicians, health providers and policy-makers.

**Table 3:** Gaps in knowledge and need for further research.

CKD clinical epidemiology among the old patients		
Lessons learned	Remaining gaps	Suggestion for action
Selection of healthier patients referred to nephrologists	Criteria for referral to a nephrologist in real-life situation. Information on outcomes of patients not referred to nephrologists.	Enhance communication and cooperation between general practitioners and nephrologists. Disseminate clear guidelines for referral to nephrologists. Develop studies in general population (laboratories, general practitioners, geriatricians.).
Complex medical regimen with frequent adverse effects and inappropriate use	Precise benefit-risk of such polypharmacy. Standardized methodology of reconciliation process of drug therapy.	Develop methodology in reconciliation process of drug therapy applicable to polypathological patients. Enhance training of health professionals on drugs delivery in this population.
Inaccuracy of existing widely-used formula to estimate renal function		Develop implementation studies on new formulas and their impact on drugs prescription
Treatment options information provided very late in the course of CKD	Communication skills of health professionals. Patients’ motivation and ability	Develop educational program for patients in each nephrological units. Evaluate gap of early personalized care plan. Develop training of general practitioners and geriatricians. Evaluate and compare models of share decision making.
High frequency of dialysis initiation in emergency on temporary access	Exact timing for vascular access preparation and dialysis initiation	Develop studies on patient’s trajectories and outcomes
High initial mortality after dialysis initiation	Expected benefits of dialysis start.	Enhance multidisciplinary care and systematic geriatric assessment.
Little conservative treatment proposal. Low access to palliative care.	Specific prognosis scores to support detailed information	Develop training of health professionals on conservative care alternatives. Enhance staff training in ethics and communication. Developp specific prognosis score for decision-aid tools. Propose common training in nephrology and palliative care.
High clinical impact of CKD stages on the physical dimensions of Qol and to a lesser extent of mental dimensions	Impact of prevention and symptoms management on Qol	Initiate generalization and systematization of Patient Reported Outcomes measurement. Design Randomized clinical trials inclusive on patient-centred outcomes
High variability in treatment access	Optimal pathways. Reasons of variability in treatment care	Evaluate of shared-decision process. Studies to quantify the impact on Qol of different treatment care
Low probability of registration and variations in kidney transplant waiting list practices	Criteria for referral to a transplant centre in real-life situation.	Enhance systematic screening of all older patients for transplantation eligibility to rapidly identifying suitable candidates. Develop decision tools capable of identifying patients with a good long- term prognosis after renal transplantation
High hospitalization rates and care costs	Impact of cardiovascular prevention. Role of readmission.	Develop health economic model. Enhance studies on readmission causes.

The transition period between Stage 4 CKD and kidney failure is crucial and has a major impact on the subsequent prognosis (76). It is therefore essential to smooth this transition by better identifying the phenotypes particularly at risk of a poor outcome on renal replacement therapy (77). An important item in the global assessment is frailty and the identification of geriatric syndromes (76, 78–80). Frailty, favored both by age and CKD, is very common in this population and is associated with multiple complications such as falls, dependence, institutionalization, recurrent hospitalizations and death (81). Several tools have been developed to assess it in practice and, in the future, it seems essential to systematically integrate these data into registries (82).

Despite the IDEAL randomized trial in the early 2010s (83), there is currently no clear answer on the right time to begin dialysis, especially in the older adults. Recent developments in the field of epidemiology and the extension of renal registries to patients with Stage 4-5 CKD may provide an answer to this question in the future (84, 85).

The complexity of managing advanced CKD (especially in the older adults) justifies the organization of care around coordinated care pathways (1, 77). In France, an annual flat-rate payment for healthcare structures was introduced in 2019 with the aim of improving the follow-up and support of CKD patients, by combatting disease progression and the occurrence of complications, whilst optimizing the transition to kidney failure around coordinated multi-professional care (https://www.legifrance.gouv.fr/loda/id/JORFTEXT000039138244/).

The particularly high frequency of emergency dialysis initiation on temporary access in older patients has been mentioned above, even in patients under close nephrology follow-up. This potentially refers to an additional option in the choice of therapeutic project: the “Deciding Not to Decide” option (86). This choice is particularly appreciated in the older adults, who prefer to focus on the present to preserve their quality of life at all costs. In order to improve the patient-centered and shared decision-making approach, it would be necessary to go further into the study of what determines the patients’ choice for their care plan. This would allow a better understanding of what is at stake at the time of the decision, especially as the health priorities of caregivers and patients with respect to advanced CKD are not aligned (87).

Kidney registries are well placed to facilitate the large-scale collection of patient-reported outcome measures (PROMs) or patient-reported experience measures (PREMs) to facilitate improvements in health care and organization (88, 89). Such collection may lead to more valuable, person-centered services and enhanced health and wellbeing of people with CKD. Although the quantitative approach does not rule out qualitative studies, registry may help to identify the population to be included in such survey (90).

To guide nephrologists in their daily practice and the patients’ decisional process, prognostic scores have an increasingly important role to play. However, the scores developed have shown limited accuracy in the older adults (47, 48). Furthermore, using mortality as the outcome of choice must be questioned in the older adults: (i) the mortality rate is very high in this population burdened by comorbidities and clinical frailty (2); older patients often place more importance on their quality of life than on life expectancy. Thus, in the coming years, with the help of registries and cohorts of CKD patients, it is important to develop approaches based on quality of life data or relevant proxies such as time spent in hospital. New prognostic scores could thus be developed for an even more patient-centered therapeutic education (91).

From a public health view point, the needs in health care are moving fast and the care offer must adapt to the most vulnerable frail patients. Therefore, registry data, in providing epidemiological knowledge, by describing specific health consumption in detail, are appropriate for better predicting the needs for suitable care arrangements i.e. facility-based, home-based or supportive care. Although an individualized approach is recommended, few studies are available to assess the benefits of alternatives to standard care. By providing real-life data, registers can provide invaluable insights. Individualized care does not rule out differences in practices between professionals that may represent a loss of opportunity for patients. Such evaluations should be made by registries to help health authorities to guarantee the equality of care throughout the territory. Furthermore, in order to offer a real alternative to renal replacement therapies for older frail patients, the deployment of conservative care requires multi-level resources within a coordinated network, supported by significant policy changes (92). Since the end of 2020, an experiment supported by the French national health insurance fund has been implemented to offer home-based medical care programs dedicated to patients undergoing conservative care (https://solidarites-sante.gouv.fr/IMG/pdf/soins_conservateurs_irct_arrete.pdf 2019). This French home-based care network now includes over a hundred patients, and its permanent legal registration is currently under consideration. Thus, CKD registries and cohorts also have an important role to play in monitoring and evaluating both the clinical and economic impact of these types of initiatives.

There is a growing role of registries in randomized controlled trials. The REIN registry is currently conducting a pilot study on the model of the ANZDATA Swift study, a Registry-Based Cluster Randomised Controlled Trial to determine the clinical effectiveness and cost-effectiveness of symptom monitoring with feedback to clinicians and patients compared with standard care in improving quality of life outcomes at 12 months for adults on haemodialysis.

(93). Embedded within a registry, randomized trials capitalize on established and secure data collection system. It is a new way to decrease research cost by avoiding extra collection of information that already exist and are available. In older patients, avoiding extra visits is alos an asset.

The linkage between registry data and health databases is an opportunity to assess expenditures and health care use. In this older population, the medication burden and inappropriate prescription risk are high (35). The linkage between registry data and health databases is an opportunity for in-depth assessment of drug use at “low cost” without the need to go back to the patient or the medical files. Moreover health economics evaluations are necessary for health authorities and health providers to be able to offer sustainable arrangements. As CKD is most often incurable, the most important area of work remains prevention. In order to better identify the target population for this prevention, linking the registry data to health insurance data could eventually allow us to better identify potential CKD patients at an earlier stage. Work on the French health insurance database is currently underway to develop an algorithm to recognize these patients, the first version of which was recently published (94).

Finally, the REIN registry, backed by its expertise and national network, was able to set up epidemiological monitoring of chronic dialysis and/or transplant patients affected by SARS-CoV-2 very quickly (95). A weekly newsletter has been set up (still on-going), sent to all nephrologists, using information transmitted by all the REIN research assistant working with the nephrologists in the regions. These data have shown the usefulness of vaccination (96). It helped the policy-makers identify priority patients for vaccination (97). But unless other sentinel surveillance system (98), it was not a real-time information, due to manual collection. However, it allowed various study that may be useful in the event of a new sanitary crisis. For example, the impacts of kidney transplantation moratorium on the life expectancy of kidney transplantation candidates was simulated. Patients older than 60 were less impacted by KT moratoriums: they had a lower additional time on the waitlist and a lower overmortality than younger patients in each of the three studied populations (99). The role of registers as potential health monitoring tools is greatly underestimated.

## Author contributions

All authors contributed to the conception and writing of the manuscript. All authors read and approved the final manuscript.
